# Integrated multi-dimensional deep neural network model improves prognosis prediction of advanced NSCLC patients receiving bevacizumab

**DOI:** 10.3389/fonc.2023.1052147

**Published:** 2023-02-14

**Authors:** Butuo Li, Linlin Yang, Chao Jiang, Yueyuan Yao, Haoqian Li, Shuping Cheng, Bing Zou, Bingjie Fan, Linlin Wang

**Affiliations:** ^1^ Department of Radiation Oncology, Shandong Cancer Hospital and Institute, Shandong First Medical University and Shandong Academy of Medical Sciences, Jinan, Shandong, China; ^2^ Department of Otorhinolaryngology Head and Neck Surgery, Shandong Provincial Hospital Affiliated to Shandong First Medical University, Jinan, Shandong, China; ^3^ Shandong Provincial Hospital, Cheeloo College of Medicine, Shandong University, Jinan, Shandong, China

**Keywords:** bevacizumab, non-squamous NSCLC, prognosis prediction, deep learning, radiomics

## Abstract

**Background:**

The addition of bevacizumab was found to be associated with prolonged survival whether in combination with chemotherapy, tyrosine kinase inhibitors or immune checkpoint inhibitors in the treatment landscape of advanced non-small cell lung cancer (NSCLC) patients. However, the biomarkers for efficacy of bevacizumab were still largely unknown. This study aimed to develop a deep learning model to provide individual assessment of survival in advanced NSCLC patients receiving bevacizumab.

**Methods:**

All data were retrospectively collected from a cohort of 272 radiological and pathological proven advanced non-squamous NSCLC patients. A novel multi-dimensional deep neural network (DNN) models were trained based on clinicopathological, inflammatory and radiomics features using DeepSurv and N-MTLR algorithm. And concordance index (C-index) and bier score was used to demonstrate the discriminatory and predictive capacity of the model.

**Results:**

The integration of clinicopathologic, inflammatory and radiomics features representation was performed using DeepSurv and N-MTLR with the C-index of 0.712 and 0.701 in testing cohort. And Cox proportional hazard (CPH) and random survival forest (RSF) models were also developed after data pre-processing and feature selection with the C-index of 0.665 and 0.679 respectively. DeepSurv prognostic model, indicated with best performance, was used for individual prognosis prediction. And patients divided in high-risk group were significantly associated with inferior PFS (median PFS: 5.4 vs 13.1 months, P<0.0001) and OS (median OS: 16.4 vs 21.3 months, P<0.0001).

**Conclusions:**

The integration of clinicopathologic, inflammatory and radiomics features representation based on DeepSurv model exhibited superior predictive accuracy as non-invasive method to assist in patients counseling and guidance of optimal treatment strategies.

## 1 Introduction

Bevacizumab, an intravenously administered monoclonal antibody targeting vascular endothelial growth factor (VEGF) pathway, has been proved to have effect on the inhibition of vascular growth, regression of newly formed vessels and normalization of vasculature, thereby facilitate the delivery of cytotoxic chemotherapy (1, 2). The pivotal ECOG4599 demonstrate the efficacy of the addition of bevacizumab to first-line standard chemotherapy in advanced non-squamous NSCLC patients, which can firstly extend the overall survival (OS) to more than 1 year for these patients (3). And the advantages of bevacizumab were also demonstrated in Chinese patient population with the median OS ranging from 17.7 to 24.3 months (4).

With the widespread use of immunotherapy and target therapy, the importance of anti-angiogenesis including bevacizumab seems to be weakened for the first-line treatment of advanced NSCLC patients. It should be noted that the direct action of anti-angiogenesis is vasculature in stroma rather than tumor cells, thus the combination treatment is necessary, and their anti-tumor effect might be magnified in several times (2). The pivotal study IMpower150 also demonstrated the clinical benefit of combination of atezolizumab, bevacizumab and chemotherapy, and the benefit was observed regardless of EGFR and ALK status (5). Thus, bevacizumab likely remains an important role in the treatment landscape for NSCLC patients in the future, especially as a partner in combination treatment strategies. Although the prognostic biomarkers for bevacizumab have been investigated before, such as hypertension, circulating parameters (6), there was still no robust applicable biomarker which is vital for the selection of optimal treatment strategies and individual treatment strategies. Based on the complexity of anti-angiogenesis, multi-dimensional features might exhibit better ability to differentiate survival risk of patients compared with single factor.

The predictive role of clinicopathological and systemic inflammation has been proved in our previous studies (7). Besides, radiomics refers to the highly throughout extraction and analysis of quantitative image features from medical images, and has been used to explore the potential relationship between clinical outcomes and biology of tumor (8). Previous studies have indicated the widespread application of radiomics in lung nodule detection, segmentation, characterization, prognosis prediction and clinical decision making (9).

Deep neural network (DNNs), a subset of artificial intelligence (AI), is an especially promising method that could automatically identify highly intricate and linear/nonlinear associations in data (10), thereby providing evaluations in a quantitative manner. In contrast to machine learning methods, DNNs can realize automated quantification and selection of features and excel at extracting complex features from high-dimensional data and images (11). DNNs have increasingly been deployed in radiomics and development of multi-dimensional models.

The Cox proportional hazard (CPH) regression, which performs the multivariate linear regression between survival time and variables, is the most common survival prediction (12). One limitation of CPH is the linear nature which might result in the neglection of nonlinear relationships between features, while DNN could excel at this task in theory. The potential advantage of DNNs, such as Cox-nnet, DeepSurv (13) and AECOX (14), has been confirmed in predicting prognosis compared to Cox-PH and traditional ML models (15). Yet, the predictive role of DNNs prognostic model based on radiomics is still unclear for advanced NSCLC patients receiving bevacizumab. This study aimed to explore the prognostic effect of radiomics features for advanced NSCLC patients receiving bevacizumab. And we proposed to develop a respective integrated DNN survival model based on three kinds of variables, and perform the comparison of performance between DNNs model and machine learning model to identify the superiority of DNN survival model.

## 2 Materials and methods

### 2.1 Patient population

Patients with advanced NSCLC who underwent bevacizumab plus standard chemotherapy in Shandong Cancer Hospital, between July 2014 and October 2019, were enrolled in this study. This study was approved by the Ethics Committee of Shandong Cancer Hospital and was conducted in accordance with the principles of the 1975 Declaration of Helsinki and its later amendments or comparable ethical standards. The inclusion criteria were as follows, (1) radiological and pathological confirmed stage IIIB-IV non-squamous NSCLC; (2) first or second line treated with bevacizumab plus standard chemotherapy for at least two cycles (3 weeks as one cycle); (3) available clinicopathological and hematological data. The exclusion criteria included (1) the synchronization application with target therapy or immunotherapy; (2) combined with other malignancies or hematologic diseases. Moreover, advanced non-squamous NSCLC patients, who met the selection criteria, from Phase III clinical trials which compared the efficacy between bevacizumab and QL1101-002 or BP102 were retrospectively enrolled into external validation cohort.

### 2.2 Acquisition of clinical and inflammatory variables

The medical records of each patient were reviewed with respect to consecutive laboratory clinical factors and complete blood count during bevacizumab treatment. All data were acquired retrospectively using uniform database templates to ensure consistent data collection. Specifically, clinical parameters included gender, age, smoking status, EGFR status, anatomical location (central or peripheral), and the presence or absence of liver, brain, or bone metastases.

Inflammatory factors included NLR, PLR, LMR and lactate dehydrogenase (LDH). NLR was defined as the ratio of absolute neutrophil count to absolute lymphocyte count; PLR was the ratio of absolute platelet count to lymphocyte count; LMR was constructed with the ratio of absolute lymphocyte count to absolute monocyte count. The dynamic changes of systemic inflammatory factors were collected during bevacizumab treatment. ROC curves were performed to confirm the cut-off of inflammatory factors.

### 2.3 Acquisition of CT images

The contrast-enhanced CT images before bevacizumab treatment were extracted from Picture Archiving and Communication Systems using a SOMATOM Definition AS (Siemens Healthineers) for each population in this study. The scanning parameters were as follows: tube voltage, 120 kVp; tube current, 200 mAs; detector, 64 × 0.625 mm; reconstruction thickness, 5 mm; reconstruction interval, 5mm. The CT images were exported and stored in the form of Digital Imaging and Communications for further analysis.

### 2.4 Patient follow‐up and outcomes

The primary endpoint was progression free survival (PFS) and the secondary endpoint was OS. Tumor response was measured according to Response Evaluation Criteria in Solid Tumors (RECIST) version 1.1. PFS and OS were defined as the time from the initiation of bevacizumab to the date of progression and the date of death or last follow-up, respectively. The last follow-up time was until December 2019.

### 2.5 Training and validation of prognostic model based on the machine learning methods

The region of interest (ROI) in each slice of CT images was defined as the primary lesion of tumor and contoured manually by two radiation oncologists using open‐source imaging biomarker explorer (IBEX) software (http://bit.ly/IBEX_MDAnderson) with the window/level of 600/1000 HU. All CT images were countered twice with the interval of about two months in order to reduce the operator bias. The 3D ROI was then preprocessed, and a set of 1041 radiomics features were extracted using IBEX software. All features were divided into nine categories including Intensity, Intensity Histogram, Shape, Gradient Direction Histogram, Gray Level Co-occurrence Matrix 25 (GLCM25), Three-Dimensional Gray Level Co-occurrence Matrix (GLCM3), Two-Dimensional Neighbor Intensity Difference (NID25), Three-Dimensional Neighbor Intensity Difference (NID3), Gray Level Run Length Matrix (GLRLM). Intra-class and Inter-class correlation coefficient (ICC) were calculated for the features extracted from the ROI delineated by two physicians, and features with ICC≥0.75 were screened as stable imaging features. 75% of patients were selected at random to be held out for radiomics feature selection, and validation was done on the remaining 25% of patients.

Univariate and multivariate Cox regression was performed for feature selection among clinicopathological and hematological inflammatory factors. LASSO-Cox analyses were performed to achieve feature dimension reduction for radiomics features. Machine learning prognostic models were performed using CPH and random survival forest (RSF) algorithm.

### 2.6 Training and validation of multi-dimensional deep neural network survival models

DNN survival models were trained with the network of DeepSurv (16) and Neural Multi-Task Logistic Regression (N-MTLR) (17), and nonlinear variation of parameters was the core of DNN. DeepSurv is a nonlinear extension of CPH model and constructed by using feedforward deep neural network and multilayer perceptron. In addition, the multilayer perceptron is used to estimate the probability of the occurrence of interested events in different time intervals and build the N-MTLR model. Various combinations of hyperparameters were explored in order to optimize the DNN, including batch size, layer of the network and number of neurons of network. The final DNN model was a balance between performance and computing cost, and assigned precise weight to each variable after training and iterations. All patients were divided into high-risk and low-risk group based on the DNN model.

**Figure 1:**
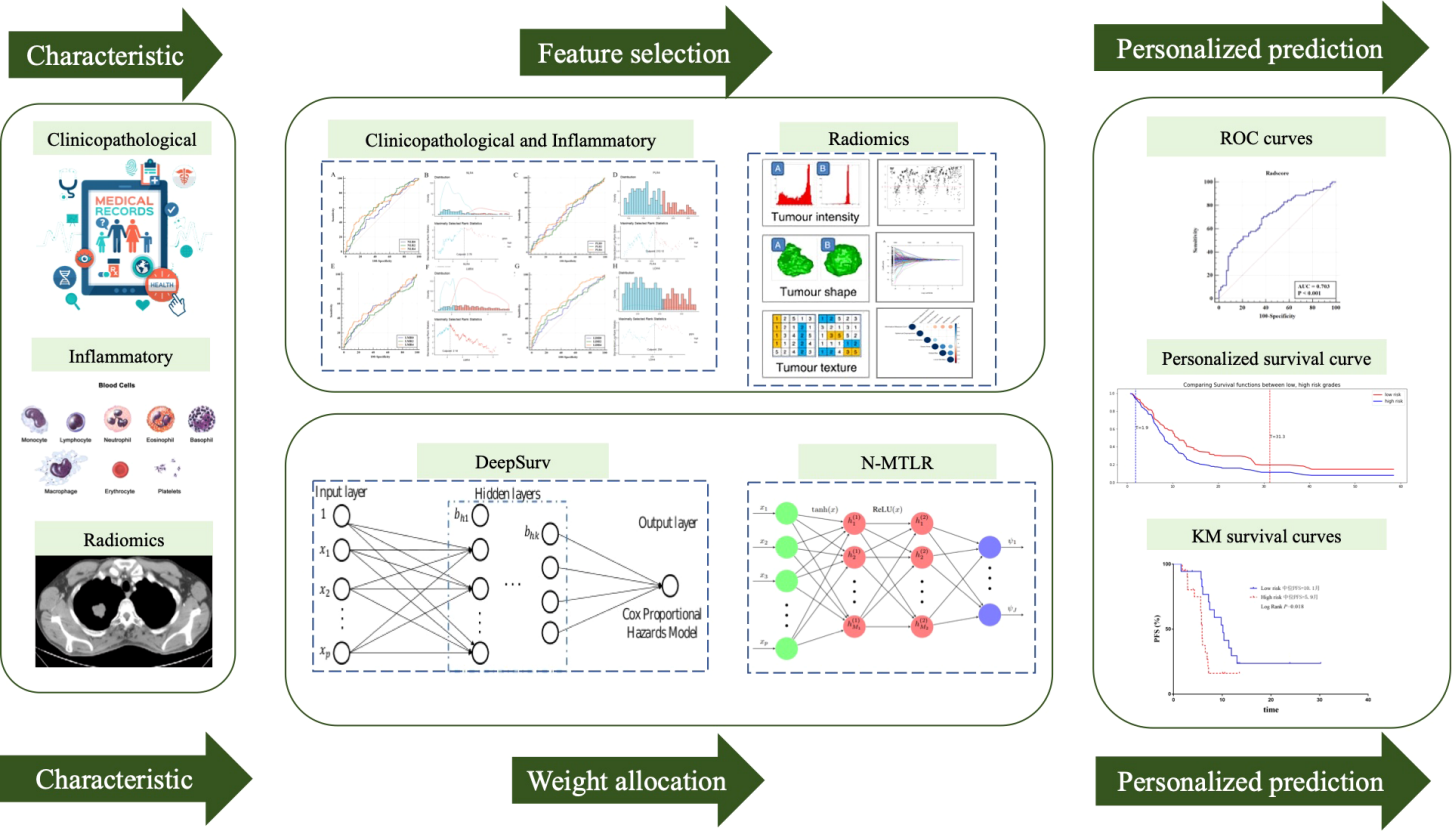
Graphic flow chart of development of prognostic model.

The clinicopathological and inflammatory variables and radiomics features were used as the input of DNN, and PFS time and PFS status were the output. The graphical flow chart of the study was shown in **Figure 1**.

### 2.7 Statistical analyses

All patients were randomly divided into training cohort and testing cohort with the split of 70% and 30% once for the train and test of prognostic models. Concordance index (C-index), integrated Brier scores (IBS) and median absolute error (MAE) were used to evaluate and compare the performances of prognostic models of different models. The IBS measures the accuracy of probabilistic predictions of models and the lower IBS indicates the accurate predictions. MBE measures the variability between predictions and realities. The evaluation of model was performed three times in testing cohort, and the median of C-index, IBS and MAE were calculated to evaluate the performance.

Kaplan-Meier survival analysis was performed to calculate median survival time and plot the survival curve, and log-rank was used to compare the survival curves. All statistical analyses are two-sided and P value less than 0.5 was considered as significant. Train of DNN is based on the “NumPy” and “SciPy” function, and PyTorch framework, and train of CPH and RSF model is based on “CoxPHModel” and “RandomSurvivalForestModel” function of python 3.6.0.

## 3 Results

### 3.1 Patient demographics and characteristics

There were 272 advanced non-squamous NSCLC patients enrolled in this study, and CT images before bevacizumab treatment were available for 195 of them. Entire 195 patients were randomly and automatically split into a training cohort and validation cohort. The baseline detail characteristics of enrolled population were shown in **Table 1**, and clinicopathological and demographic parameters were balanced between two cohort of patients. 147 patients were included in training cohort, and the median PFS and OS were 8.3 and 26 months respectively.

**Table 1:** Patient Demographics and Characteristics of 195 patients.

Parameters	Training cohort (%)	Validation cohort (%)	P
Age			0.90
≤57	72 (49%)	24 (50%)	
>57	75 (51%)	24 (50%)	
Gender			0.76
Male	82 (55.8%)	28 (58.3%)	
Female	65 (44.2%)	20 (41.7%)	
Smoking History			0.33
No	100 (68%)	29 (60.4%)	
Yes	47 (32%)	19 (39.6%)	
Anatomical type			0.56
Peripheral	107 (72.8%)	37 (77.1%)	
Central	40 (27.2%)	11 (22.9%)	
EGFR			0.31
Wild type	78 (53.1%)	30 (62.5%)	
Sensitive mutation	51 (34.7%)	10 (20.8%)	
Resistance mutation	5 (3.4%)	3 (6.3%)	
NA	13 (8.8%)	5 (10.4%)	
Bone metastasis			0.94
No	94 (63.9%)	31 (64.6%)	
Yes	53 (36.1%)	17 (35.4%)	
Brain metastasis			0.87
No	106 (72.1%)	34 (70.8%)	
Yes	41 (27.9%)	14 (29.2%)	
Liver metastasis			0.85
No	127 (86.4%)	42 (87.5%)	
Yes	20 (13.6%)	6 (12.5%)	

### 3.2 Training and validation of prognostic model based on the conventional machine learning methods

#### 3.2.1 Clinicopathological and hematologic inflammatory characteristics preprocessing

There were 220 patients experienced disease progression among 272 patients, and the median PFS was 8.2 months. ROC curves of NLR, PLR, LMR and LDH based on 6-month PFS illustrated that hematologic inflammatory characteristics after 4 cycles treatments were the most predictive, which were included in further feature selection. According to the ROC curves, the value with the maximum Youden index was selected as the cut-off value, and the cut-off values of NLR4, PLR4, LMR4 and LDH4 were set as 2.78, 212.1, 2.18 and 256, respectively (**Supplementary Figure 1**). All patients were divided into high and low groups.

Univariate and multivariate Cox analysis indicated that smoking history (HR=1.72, P=0.001), anatomical type (HR=1.95, P<0.0001), bone metastasis (HR=1.45, P=0.025), liver metastasis (HR=1.52, P=0.056), NLR4 (HR=1.98, P<0.0001) and LDH4 (HR=1.84, P<0.0001) were the independent prognostic factors for bevacizumab (**Table 2**).

**Table 2:** Univariate and multivariate cox analysis of 272 patients.

Parameters	Uni-HR	95% CI	P	Multi-HR	95% CI	P
Age	1.2	0.94-1.6	0.13			
Gender	0.8	0.61-1	0.1			
Smoking History	1.9	1.4-2.5	<0.0001	1.72	1.25-2.37	0.001
Anatomical type	1.3	1.2-1.5	<0.0001	1.95	1.42-2.68	<0.001
EGFR			0.15			
Sensitive mutation	0.74	0.55-1.1	0.057			
Resistance mutation	1.02	0.53-1.9	0.96			
Bone metastasis	1.3	0.97-1.7	0.087	1.42	1.05-1.92	0.025
Brain metastasis	0.94	0.7-1.3	0.67			
Liver metastasis	2.1	1.4-3	0.00012	1.52	0.99-2.34	0.056
NLR4	2.3	1.6-3.1	<0.0001	1.98	1.42-2.74	<0.001
PLR4	1.4	1-1.9	0.031			
LMR4	0.59	0.44-0.8	0.001			
LDH4	1.9	1.4-2.6	<0.0001	1.84	1.32-2.57	<0.001

#### 3.2.2 Radiomic feature pre-processing

**Figure 2:**
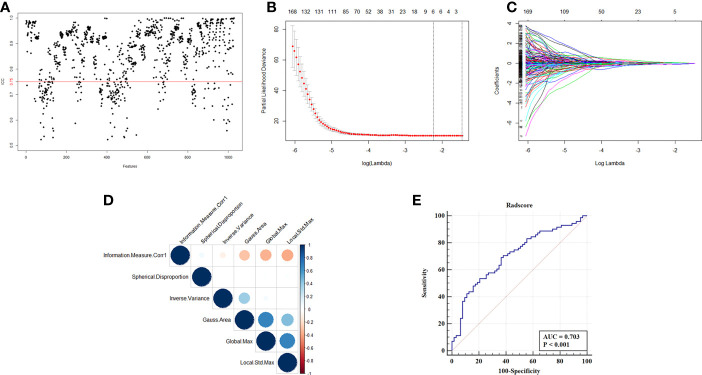
Selection of radiomics features and construction of Radscore. **(A)** ICC map of radiomics feature from two independent radiologist. **(B)** Partial likelihood deviance of radiomics features revealed by the LASSO-Cox regression model. The red dots represented the partial likelihood of deviance values, the gray lines represented the standard error (SE), the two vertical dotted lines on the left and right represented optimal values by minimum criteria and 1-SE criteria, respectively. Minimum criteria were used to select host radiomics features in model. **(C)** LASSO coefficient profiles of the survival-related radiomics features. **(D)** Correlation analysis of radiomics features selected by LASSO-Cox regression model. **(E)** ROC curve of Radscore with the AUC of 0.703.

Total of 1041 radiomic features were extracted from patients in training cohort. In order to ensure the stability and reproducibility, 740 radiomic features with ICC more than 0.75 were considered as stable features and used in following analyses (**Figure 2A**). LASSO-Cox analyses were performed to achieve feature dimension reduction (**Figure 2B**). The model exhibited the optimal performance and the least number of independent variables with the log λ=0.107 (**Figure 2C**). As the values of λ increased, the LASSO coefficients of these variables were close to zero. As a result, six radiomic features were utilized for the establishment of a prognostic signature. The correlation analysis was further performed to reduce the redundant feature. Finally, Information Measure Corr1, Inverse Variance, Local Std Max, Gauss Area and Spherical Disproportion were selected based on Lasso-Cox analysis (**Figure 2D**). The Radscore of each patient was calculated according to the weight coefficients of these 5 independent features, with the C-index of 0.65 and AUC of 0.703 after internal validation. (**Figure 2E**). The result of multivariate cox analysis also showed that the Radscore was the independent prognostic factor for NSCLC patients receiving bevacizumab whether in training cohort and validation cohort (**Supplementary Table 1** and **Supplementary Table 2**).

#### 3.2.3 Training and validation of CPH and RSF model

**Figure 3:**
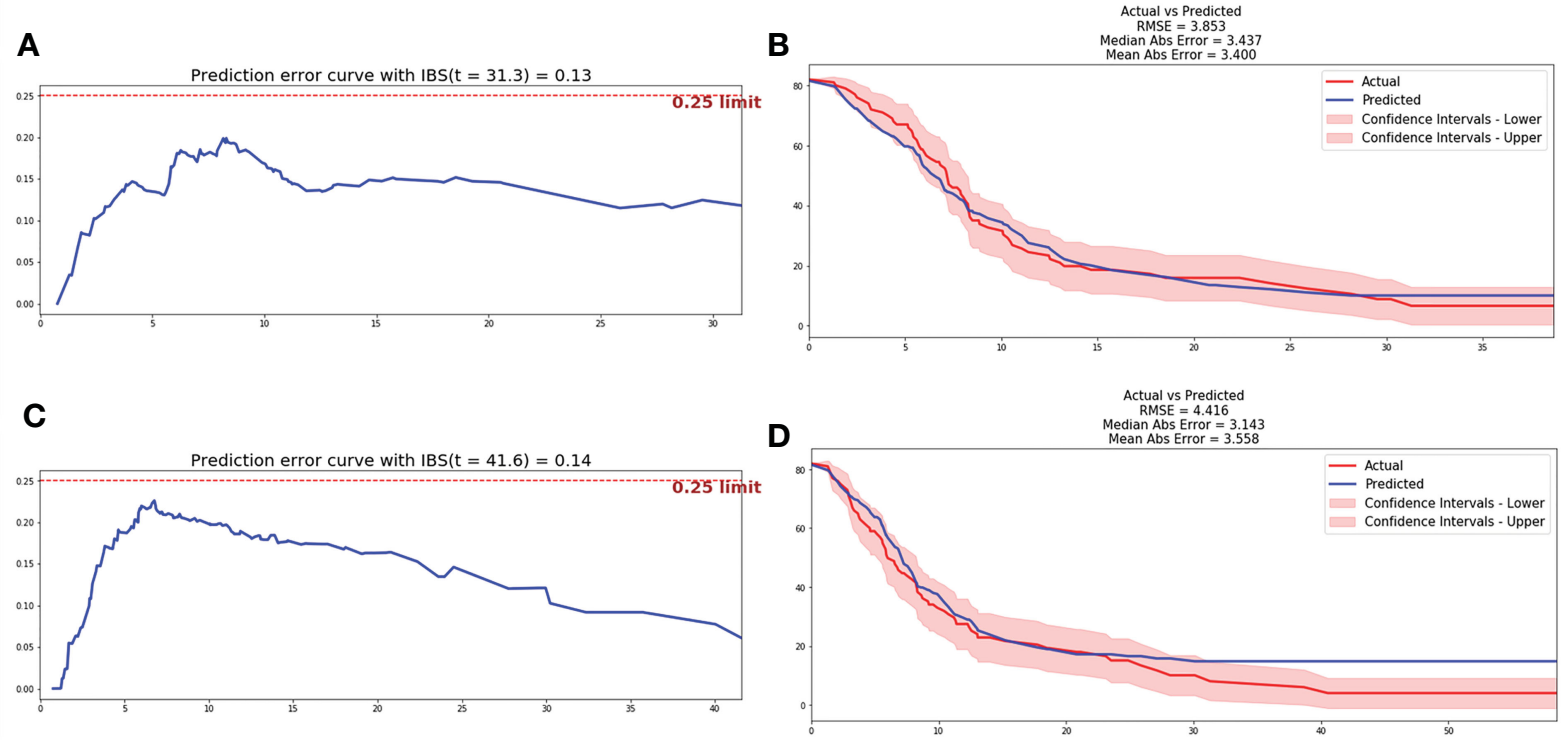
Prediction error curve and correction curve of CPH and RSF model. **(A)** Prediction error curve of CPH model. **(B)** Prediction error curve of RSF model. **(C)** Correction curve of CPH model. **(D)** Correction curve of RSF model.

After data pre-processing, smoking history, anatomical type, bone metastasis, liver metastasis, NLR4, LDH4 and Radscore were indicated to be the independent prognostic factors of bevacizumab, and included in the low dimensional feature set. And the CPH model and RSF model were trained in training cohort and estimated in testing cohort. The C-index of CPH prognostic model was 0.665, IBS was 0.13 and MAE was 3.4. The RSF model was trained on the training set with 10,000 trees and the maximum depth of the survival tree is 10. Prediction accuracy was then measured on the test set, with the C-index of 0.679, IBS of 0.14 and MAE of 3.56. The prediction error curve and calibration curve between predicted and actual survival were shown in **Figure 3**.

#### 3.2.4 Training and validation of prognostic model based on the deep learning methods

**Figure 4:**
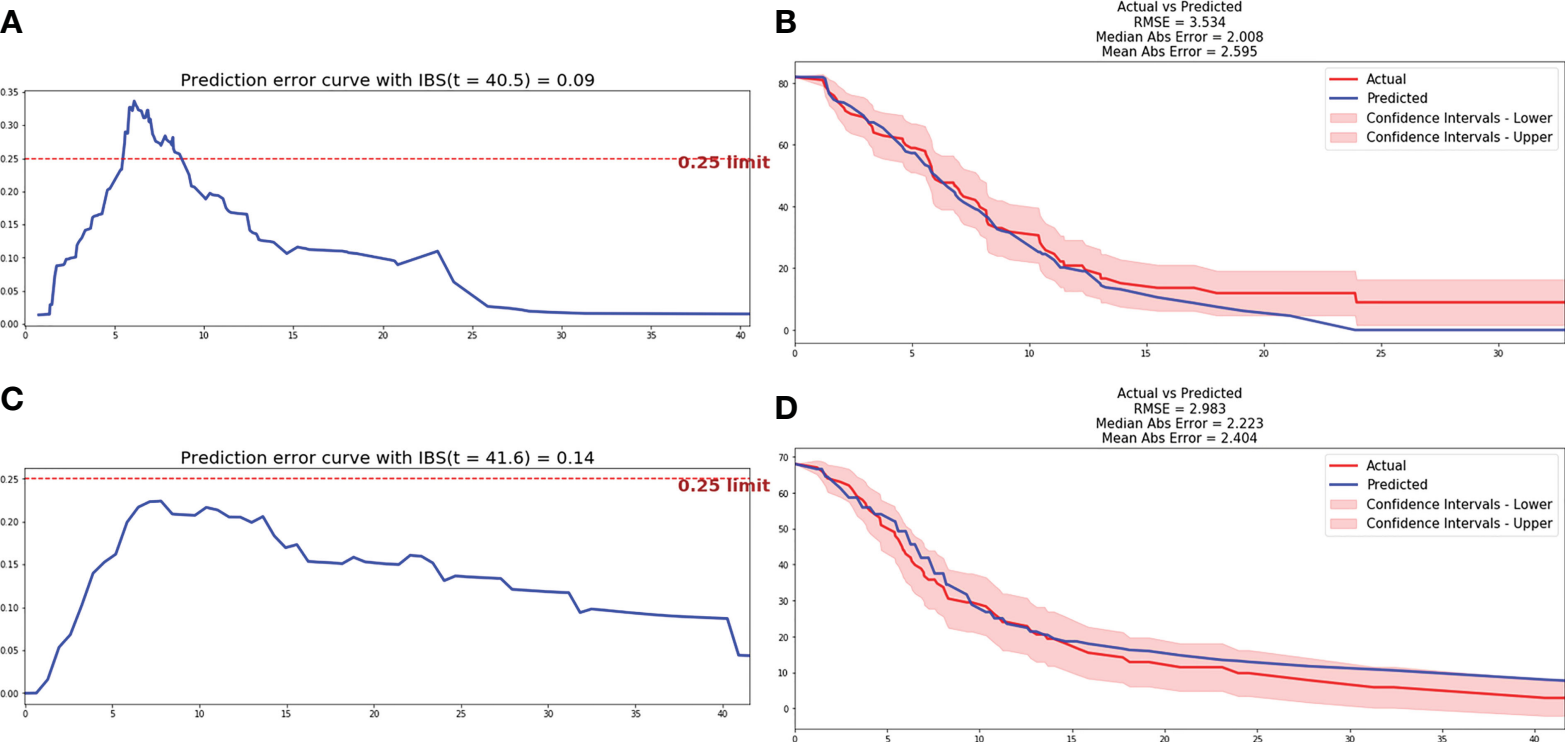
Prediction error curve and correction curve of DeepSurv and N-MTLR model. **(A)** Prediction error curve of DeepSurv model. **(B)** Prediction error curve of DeepSurv model. **(C)** Correction curve of N-MTLR model. **(D)** Correction curve of N-MTLR model.

Based on the automatic learning characteristics, optimal weight of parameters could be obtained based on deep learning methods, and feature selection was not necessary. DeepSurv and N-MTLR prognostic models were trained based on all parameters included in clinicopathological, inflammatory, and radiomics characteristics. The structure of the final model included one input layer, four hidden layers, and one output layer, and each hidden layer has 100 neurons. Hyperparameters were adjusted in order to achieve the prognostic model with best performance. The He_uniform initialization method applicable to ReLu activation functions was used to initialize weight parameters. Batch normalization was performed to reduce internal variance deviation. Dropout and L2 regularization were applied to reduce overfitting, and the dropout rate was 0.2. Adam optimizer based on gradient was used to obtain stable convergence. After 1000 iterations, the loss value achieved stability gradually, and the final prognostic model was a balance between performance and computing cost. Finally, the C-index of 0.712 and 0.701 was achieved for DeepSurv and N-MTLR prognostic model respectively in testing cohort. The IBS was 0.09 and 0.14, and MAE was 2.6 and 2.4 for DeepSurv and N-MTLR respectively. The prediction error curve and calibration curve between predicted and actual survival of deep learning prognostic models were shown in **Figure 4**.

**Figure 5:**
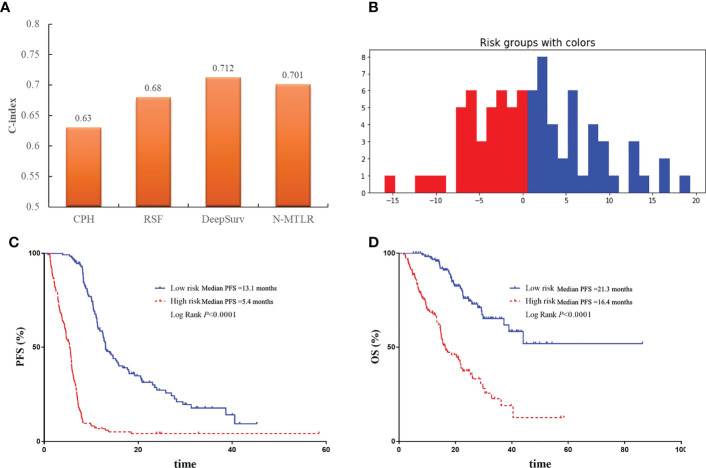
The comparison and validation of the performance of DeepSurv models. **(A)** The comparison of performance of machine learning and deep learning models. **(B)** All patients were divided into high-risk and low-risk group according to DeepSurv model. **(C)** Comparison of Kaplan-Meier survival curves of PFS between high-risk and low-risk patients. **(D)** Comparison of Kaplan-Meier survival curves of OS between high-risk and low-risk patients.

The comparison of performance between machine learning and deep learning prognostic model was illustrated in **Figure 5A**. All patients were divided into high-risk and low-risk group based on DeepSurv prognostic model (**Figure 5B**), and the performance was further validated with Kaplan-Meier curves. Patients in high-risk group were significantly associated with inferior PFS (median PFS: 5.4 vs 13.1 months, P<0.0001) and OS (median OS: 16.4 vs 21.3 months, P<0.0001) compared to patients in low-risk group (**Figure 5C, D**).

#### 3.2.5 Validation of DeepSurv model in external validation cohort

**Figure 6:**
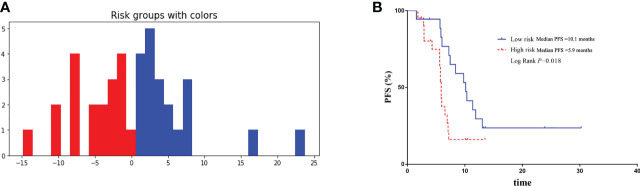
Validation of the performance of DeepSurv models in external validation cohort. **(A)** Patients in external validation cohort were divided into high-risk and low-risk group. **(B)** Comparison of Kaplan-Meier survival curves of PFS between high-risk and low-risk patients.

There were 39 advanced non-squamous NSCLC patients from Phase III clinical trials receiving bevacizumab or QL1101-002 or BP102 were enrolled into external validation cohort, and median PFS was 7.1 months (95%CI 5.7-8.5 months). Baseline characteristics were balanced between patients in retrospective and external validation cohort (**Table 3**). And the Deepsurv model also performed well in external validation cohort, with the C-index of 0.73 and the IBS of 0.15. Patients were also divided into high-risk and low-risk group. The median PFS was 10.1 months of high-risk patients and was significantly superior to patients in low-risk group, which illustrated the external applicability and generalization ability of DeepSurv prognostic model (**Figure 6**).

**Table 3:** Comparison of baseline characteristics of patients in retrospective and external validation cohort.

Parameters	Retrospective cohort (%)	External Validation cohort (%)	P
Age			0.54
≤57	139 (51.1%)	22 (56.4%)	
>57	133 (48.9%)	17 (43.6%)	
Gender			0.50
Male	155 (57%)	20 (51.3%)	
Female	117 (43%)	19 (48.7%)	
Smoking History			0.12
No	175 (64.3%)	20 (51.3%)	
Yes	97 (35.7%)	19 (48.7%)	
Anatomical type			0.16
Peripheral	194 (71.3%)	32 (82.1%)	
Central	78 (28.7%)	7 (17.9%)	
EGFR			0.26
Wild type	155 (57%)	16 (41%)	
Sensitive mutation	81 (29.8%)	16 (41%)	
Resistance mutation	11 (4%)	3 (7.7%)	
NA	25 (9.2%)	4 (10.3%)	
Bone metastasis			0.91
No	170 (62.5%)	24 (61.5%)	
Yes	102 (37.5%)	15 (38.5%)	
Brain metastasis			0.15
No	193 (71%)	32 (82.1%)	
Yes	79 (29%)	7 (17.9%)	
Liver metastasis			0.15
No	236 (86.8%)	37 (94.8%)	
Yes	36 (13.2%)	2 (5.1%)	

## 4 Discussion

In this study, we initially demonstrated the predictive role of radiomics for the prognosis of advanced NSCLC patients receiving bevacizumab. More importantly, a robust DNN prognostic model was developed using DeepSurv and N-MTLR algorithm respectively, which could be conveniently used by physicians for accurate prognosis prediction and development of individual treatment strategies for advanced NSCLC patients.

Radiomics is an emerging field in quantitative imaging, and has been widespread adopted in diagnosis, staging and evaluation of clinical outcomes for cancer patients, which might have fantastic application prospects in personalized medicine (8). Radiographic phenotypes were found to be capable of representing the underlying pathophysiology and microenvironment of tumor (18), and thus were more suitable for predicting prognosis and therapeutic response of bevacizumab which acts on the vasculature of tumor. This study firstly indicated the prognostic role of radiomics for bevacizumab treatment in NSCLC patients by development of Radscore using Lasso-Cox. And the Radscore was also found to be the independent factor of bevacizumab.

However, the predictive ability of single type of variables were limited, and clinicopathological and systemic inflammation were also included to develop a prognostic model in this study. CPH and RSF is by far the most commonly used method for survival analysis. While, the setting outcomes of these methods were the linear fitting of covariates, whose intrinsic complex nonlinearities were largely ignored (19). With the extensive application of artificial intelligence in cancer research process, DNN provide a new prospective on non-linear prognostic model by construction of complex correlations through multiple hidden layers. The DNN has been widely used in clinical and translational cancer research including diagnosis, staging, evaluation of efficacy and adverse effect (20, 21). Hosny A et al. have trained a CNN for automatic quantification and feature selection, thereby to prediction the 2-year OS of NSCLC patients. And the results indicated the reliable prediction performance of CNN with an AUC of 0.70, better than that of RSF, and were also robust in independent external datasets (22). However, the survival outcomes of this study were dichotomous variable rather than the survival or censor time, which cause the loss of outcome information and might contribute to the reduction of the prediction accuracy.

This study firstly trained the DNN prognostic model applying DeepSurv and N-MTLR for prognosis prediction of bevacizumab, whose output variables were set as PFS time and status. And the input layer of DNN consisted of clinicopathological, systemic inflammation, which has been demonstrated to be related to the efficacy of bevacizumab before (7), and radiomics variables. Besides, feature selection is not necessary as weight allocation has been performed for all parameters. DeepSurv takes the top layer of hidden layer as the input variable of proportional hazards model (13), and N-MTLR builds multi-layer neural network at different time intervals to evaluate the probability of event occurrence (17). The result indicated the best performance of DeepSurv model with the C-index of 0.712 compared to CPH and RSF models. The robust performance of DeepSurv model was also validated in external dataset, which indicated the extensive generalization and applicability of DNN model for prognostic prediction of bevacizumab. Our results indicated the superiority of DNN in prognosis prediction, especially in the analysis of high-dimensional features. It can be seen that DNN prognosis prediction is not only theoretically feasible but also can be extended to clinical practice to assist decision-making.

The prognostic biomarkers of bevacizumab were still inconclusive for advanced NSCLC patients. Our previous studies have found the predictive value of clinicopathological and systemic inflammatory factors for bevacizumab in NSCLC patients (7, 23). This study also indicated the prognostic value of radiomics features. Compared to single factor, the integrative model contains more information and can achieve better prediction performance. Thus, we developed DeepSurv prognostic model, which can be conveniently used by clinicians before and during treatment. For patients in high-risk group, another treatment strategy such as immunotherapy or combination treatment might be selected.

Although our study had many strengths, several limitations should be addressed here. Firstly, the sample size was still small, which might inevitably limit the performance and stability of DNN. Secondly, although thousands of variables were included in this study, they were still uncomprehensive and genomics, histological feature and others were not included. Thus, large cohort with more comprehensive variables are needed to optimize the DNN. Besides, DNNs are still more or less a kind of “black box” which could automatically modulate the weights of every variable upon the outcome, the potential biological mechanism needed to be further investigated in future studies.

## 5 Conclusions

Integrative DNN prognostic model was initially developed by combining radiomics signature with clinicopathological and inflammatory feature using DeepSurv. The superior performance and robustness of DeepSurv model were observed, which open up prospects for the cross disciplines between AI and survival analysis of cancer patients. This easy-to-operated DNN model could not only assist in personalized treatment and surveillance strategies, but also provide patient consultation services, and was strongly suggested to be widely applied in clinical practice.

## Data availability statement

The raw data supporting the conclusions of this article will be made available by the authors, without undue reservation.

## Ethics statement

The studies involving human participants were reviewed and approved by Ethics Committee of Shandong Cancer Hospital. Written informed consent for participation was not required for this study in accordance with the national legislation and the institutional requirements.

## Author contributions

(I) Conception and design: LW, BL and CJ. (II) Administrative support: YY, BZ and BF. (III) Provision of study materials or patients: LY, HL and SC. (IV) Collection and assembly of data: BL and CJ. (V) Data analysis and interpretation: LW and BL. All authors contributed to the article and approved the submitted version.
