# Supplementary material for: Integrated multi-dimensional deep neural network model improves prognosis prediction of advanced NSCLC patients receiving bevacizumab
Source: Front Oncol. 2023 Feb 14;13:1052147. doi: 10.3389/fonc.2023.1052147 (PMC9972089; doi:10.3389/fonc.2023.1052147)
Supplement: Supplementary file 1 [file DataSheet_1.docx]

Supplementary Material

# Supplementary Tables

**Supplementary Table 1. Univariate cox analysis in training and validation cohort of 195 patients.**

| Parameters | Training cohort (%) | | | Validation cohort (%) | | |
| --- | --- | --- | --- | --- | --- | --- |
|  | Uni-HR | 95% CI | P | Uni-HR | 95% CI | P |
| Age | 1.3 | 0.94-1.9 | 0.11 | 1 | 0.54-2 | 0.9 |
| Gender | 1.3 | 0.93-1.9 | 0.12 | 0.93 | 0.48-1.8 | 0.84 |
| Smoking History | 2 | 1.3-2.9 | 0.00072 | 2.4 | 1.2-4.8 | 0.011 |
| Anatomical type | 0.68 | 0.46-1 | 0.052 | 0.43 | 0.2-0.91 | 0.027 |
| EGFR |  |  | 0.10 |  |  | 0.86 |
| Sensitive mutation | 0.65 | 0.43-0.97 | 0.033 | 1.04 | 0.48-2.29 | 0.92 |
| Resistance mutation | 0.80 | 0.32-2.00 | 0.64 | 0.68 | 0.16-2.95 | 0.61 |
| Bone metastasis | 1.2 | 0.8-1.7 | 0.43 | 1 | 0.5-2.0 | 0.97 |
| Brain metastasis | 0.84 | 0.56-1.3 | 0.39 | 1.3 | 0.67-2.7 | 0.4 |
| Liver metastasis | 1.7 | 1-2.8 | 0.038 | 3.7 | 1.5-9.2 | 0.004 |
| Radscore | 8.5 | 4.1-18 | <0.001 | 5.5 | 1.5-20 | 0.011 |

**Supplementary Table 2. Multivariate cox analysis in training and validation cohort of 195 patients.**

| Parameters | Multi-HR | 95%CI | P |
| --- | --- | --- | --- |
| Training cohort |  |  |  |
| Smoking history | 1.83 | 1.21-2.78 | 0.005 |
| Liver metastasis | 2.02 | 1.22-3.33 | 0.006 |
| Radscore | 10.54 | 4.84-22.58 | <0.001 |
| Validation cohort |  |  |  |
| Smoking history | 3.2 | 1.51-6.80 | 0.002 |
| Anatomical type | 3.05 | 1.35-6.89 | 0.007 |
| Liver metastasis | 3.35 | 1.31-8.59 | 0.012 |
| Radscore | 4.27 | 1.06-17.3 | 0.041 |

# Supplementary Figures


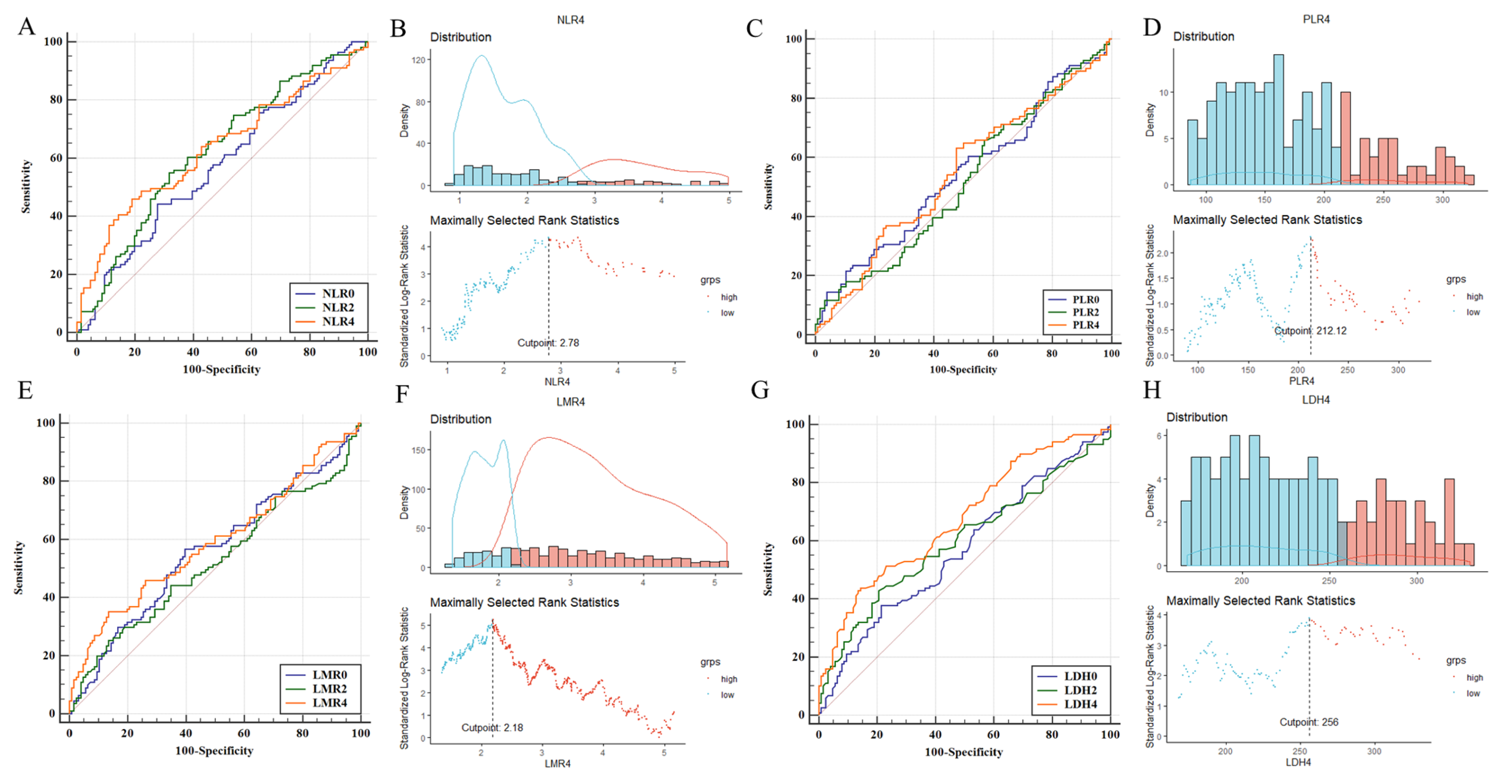


**Supplementary Figure 1.** Comparison of ROC curves of systemic inflammatory factors and the selection of optimal cut-off value. A. Comparison of ROC curves of NLR. B. Selection of optimal cut-off value of NLR4. C. Comparison of ROC curves of PLR. D. Selection of optimal cut-off value of PLR4. E. Comparison of ROC curves of LMR. F. Selection of optimal cut-off value of LMR 4. G. Comparison of ROC curves of LDH. H. Selection of optimal cut-off value of LDH 4.
